# Hydrogel carrier with bubble vibration enhancer for ultrasound-triggered drug release

**DOI:** 10.1016/j.ultsonch.2024.107173

**Published:** 2024-11-22

**Authors:** Ryuto Yamakawa, Hiroaki Onoe, Yuta Kurashina

**Affiliations:** a Division of Advanced Mechanical Systems Engineering, Tokyo University of Agriculture and Technology, 2-24-16 Nakacho, Koganei-shi, Tokyo 184–8588, Japan; b Faculty of Science and Technology, Keio University, 3-14-1 Hiyoshi, Kohoku-ku, Yokohama 223-8522, Japan

**Keywords:** Ultrasound, Drug release, Hydrogel, Acoustic responsiveness, Biocompatibility

## Abstract

Hydrogel-based drug carriers provide on-demand drug release via external stimuli. Ultrasound is a promising method because of the potential for remotely releasing the drug. However, intense ultrasound irradiation has been required in previous studies. This paper reports drug model release from hydrogel carriers encapsulating bubble vibration enhancers (BVEs) consisting of microbubbles coated with a lipid membrane. Vibration of BVEs induced by ultrasound stimulation promoted the release of drug models with ultrasound irradiation controlled to a biologically safe acoustic pressure based on spatial-peak temporal-average intensity (*I_SPTA_*). The release ratio increased significantly from 2.3 % without BVEs and ultrasound to 10.2 % with both. To evaluate the frequency response, the release ratio was measured at three different ultrasound frequencies (0.3, 1.8, and 2.5 MHz), showing increased efficiency as the frequency approached the resonance frequency of the BVEs. For *in vivo* applications, hydrogel microspherical carriers with BVEs achieved a 12 % release ratio. Poly-L-lysine coating successfully suppressed the drug release to 0.2 %. The carriers demonstrated repeated responsiveness when ultrasound was applied in three 5-minute intervals. The hydrogel carrier encapsulating BVEs we proposed is a promising *in vivo* device capable of releasing drugs on demand by ultrasound irradiation based on its high biosafety and acoustic responsiveness.

## Graphical abstract

**Figure:**
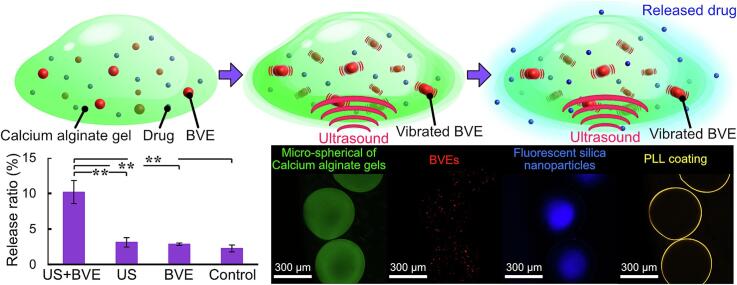

## 1 Introduction

Drug delivery systems (DDS) represent a technological advancement in the multidisciplinary field of medicine and engineering, facilitating the targeted delivery of drugs to target tissues [1]. In contrast to conventional systemic administrating methodologies [2], DDS enables drugs to work locally, thereby reducing adverse effects and facilitating more efficacious administration [3]. In the DDS, stimuli-responsive DDS, which releases drugs in response to external stimuli including heat [4], near-infrared ray [5], magnetic field [6], and ultrasound [7], has recently attracted attention as a method to control the release of DDS. These external stimuli induce structural changes in drug carriers such as micelles [8], liposomes [9], and hydrogels [10], thereby controlling the sustained release of the drug. In these materials that release drugs in response to external stimuli, hydrogel carriers with calcium alginate gel [11] and collagen gel [12], have a three-dimensional network structure with a polymer network, which can carry drugs inside the network. Furthermore, the advantage of drug release from the hydrogel by the external stimulus of the target tissue in the body is that the drug release capability is controlled on demand by applying any irradiation intensity to the drug carrier. The hydrogel carriers tend to release drugs more gradually than drugs released in other drug carriers, such as micelles or liposomes. Therefore, the capability of repeated sustained release of drugs on demand also reduces the number of drugs administrated and reduces the burden on the patient [13]. This means controlled and sustained drug release offers enormous advantages over conventional systemic administration methods, like insulin administration to diabetic patients [14] and viral gene delivery [15]. Among these stimulus-responsive DDS with hydrogel carriers, DDS equipped with acoustic responsiveness working by ultrasound stimulation has attracted particular attention due to the excellent directionality and transmissivity of ultrasound [16]. These characteristics provide appropriate stimulus irradiation to any target tissue of the body. For improvement of acoustic responsiveness, previous studies have attempted to utilize cavitation [17] occurring in the low-frequency band and to use inclusions, taking advantage of the reflection efficiency of ultrasound [18]. However, achieving acoustic responsiveness and biocompatibility with conventional hydrogel carriers is problematic regarding acoustic intensity and cavitation generation.

**Fig. 1:**
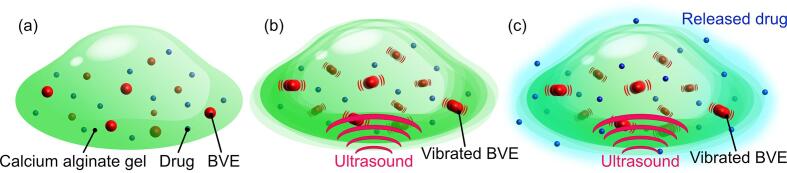
Concept of hydrogel drug release carrier having acoustic responsiveness with biocompatible bubble vibration enhancers (BVEs). (a) BVEs and drugs were encapsulated in the hydrogel carrier. (b) BVEs in the hydrogel carrier were promoted to vibrate by ultrasound. (c) Drugs were released from the hydrogel carrier because of the vibration of BVEs.

Here, we propose a DDS equipped with acoustic responsiveness with biocompatible bubble vibration enhancers (BVEs) consisting of microbubbles coated with a lipid membrane as a drug release enhancer with a vastly different acoustic impedance compared to biological tissue and hydrogel (Fig. 1). By using the resonance of BVEs, a hydrogel carrier with excellent biocompatibility is constructed by ultrasound irradiation in a frequency band with low risk of cavitation, causing biological damage. Considering the damage to the body in this study, the drug release was evaluated employing several MHz ultrasound frequencies at acoustic pressures compatible with *in vivo* and commonly used in medical ultrasound therapy. In particular, a drug model and BVEs were encapsulated in calcium alginate hydrogels. Calcium alginate hydrogels were fabricated in sheet form to evaluate the efficacy of ultrasound stimulation and BVEs by comparing the release of drug models and showing resonance vibrational characteristics of BVEs by ultrasound irradiation at varying frequencies. We finally demonstrated the applicability of the drug-releasing capability of this system by fabricating hydrogel microspherical carriers to confirm the effectiveness of BVEs and ultrasound stimulation.

## 2 Materials and methods

### 2.1 Construction of ultrasound irradiation device

The ultrasound irradiation device consists of a transducer with a piezoelectric element bonded to a glass plate and sandwiched between a felt and a silicone rubber. By bonding a glass plate to the piezoelectric element, the thickness of the transducer greatly contributed to the vibration characteristics in the thickness mode. By changing the thickness of the glass plate, the resonance frequency was varied. This structure, which is sandwiched between the felt and the silicone rubber, keeps the vibration characteristics free from the outside of the device. A polycarbonate cover was also used to hold the felt and silicone rubber, and the entire device was tightened with bolts. The resonance frequency of the fabricated ultrasound irradiation device was measured using an impedance analyzer (FRA51615, NF Corporation, Yokohama, Japan). The impedance analyzer was configured to measure impedance (Ω) and phase (deg) with an applied voltage of 2.0 V, a sweep frequency range of 10 kHz to 5 MHz, and a sweep resolution of 5000 linear steps. During ultrasound irradiation, the ultrasound frequency and applied voltage were controlled by a function generator (WF1974, NF Corporation, Yokohama, Japan), and the input voltage was increased by a kHz amplifier (HSA 4051, NF Corporation, Yokohama, Japan) and a MHz amplifier (LZY-22+, Mini-Circuits, NY, US). Furthermore, proper ultrasound irradiation was verified by real-time monitoring of voltage and current using an oscilloscope (TBS2074B, Tektronix, OR, US). The voltage was measured using a passive voltage probe (TPP1000, Tektronix, OR, US), and the current was measured using an AC current probe (CT2, Tektronix, OR, US). Acoustic pressure was measured using a hydrophone (HNR-0500, Onda Corporation, CA, US). Acoustic pressure distribution was measured at 5 mm intervals over a range of ± 15 mm from the center of the dish (430165, Corning Incorporated, AZ, US). The relationship between current and acoustic pressure was measured between 0.2 A and 1.0 A at the center of the dish. Finally, during ultrasound irradiation, the ultrasound irradiation device was placed in ice water to prevent drug model release from the hydrogel carriers caused by heat generated from the device [19]. Note that current and acoustic pressure were expressed as peak-to-peak values.

### 2.2 Fabrication of hydrogel disk-shaped carriers

As a hydrogel career, calcium alginate gel, which is highly biocompatible with flexibility and fluidity like biological tissue [20], was used. Calcium alginate gels were prepared by reacting sodium alginate (1 % (w/v), 194–13321, FUJIFILM Wako Pure Chemical Corporation, Osaka, Japan) with 100 mM calcium chloride (038–24958, FUJIFILM Wako Pure Chemical Corporation, Osaka, Japan) solution. To enhance the acoustic responsiveness of the hydrogel carriers, sonazoid® (7290414D1038, GE HealthCare Pharma, Tokyo, Japan) was included with a drug model in the hydrogel carrier as BVEs. As a drug model, fluorescent silica nanoparticles (FSNs) of 20 nm (41–00-201, micromod Partikeltechnologie GmbH, Rostock, Germany) were chosen for release demonstrations of the proposed system. In the fabrication of hydrogel disk-shaped carriers, a thin layer of sodium alginate was spread on a dish (430165, Corning, NY, US) using a spin coater (MS-B100, Mikasa Co., Ltd., Tokyo, Japan) to form a gelatinized thin film. The concentration of sodium alginate in the gelatinized thin film preparation was 1 % (w/v). The calcium alginate gel was then formed on the thin film in the dish to form hydrogel disk-shaped carriers closely adhering to the dish.

### 2.3 Observation and evaluation of BVEs encapsulated in hydrogel carriers

BVEs encapsulated in hydrogel carriers were observed with a phase-contrast microscope (ECLIPSE Ts2R, Nikon Instruments Inc., Tokyo, Japan). Additionally, BVEs were fluorescently stained with DilC_18_(5) (DiD,127274–91-3, Setareh Biotech, LLC, OR, US) and obtained Z-stack images by a confocal microscope (ECLIPSE Ti2, AX, Nikon Instruments Inc., Tokyo, Japan). The obtained confocal images of BVEs were analyzed using ImageJ (National Institutes of Health, MD, US) to measure the average particle size of BVEs.

### 2.4 Fabrication of hydrogel microspherical carriers

To fabricate hydrogel microspherical carriers by centrifugation method [21]. The tip of the glass capillary (G-1.5, NARISHIGE, Tokyo, Japan) used in the centrifugation method was pulled by a puller (PC-100, NARISHIGE, Tokyo, Japan). The tip diameter of the glass was adjusted to 70 µm by a microforge (MF-900, NARISHIGE, Tokyo, Japan). Sodium alginate (1 % (w/v)) was mixed with BVEs (2 % (v/v)) and FSNs (5 % (v/v)). The mixed solution was placed in a glass capillary for 16 µL. It was drained by a centrifuge (H-19α, KOKUSAN Co.Ltd., Saitama, Japan) into a 100 mM calcium chloride solution filled in a 1.5 mL microtube (1–7521-01, AS ONE Corporation, Osaka, Japan) to form hydrogel microspherical carriers. The fabricated hydrogel microspherical carriers were then washed with 5 mM calcium chloride solution and immersed in poly-L-lysine (PLL) (25988–63-0, Sigma-Aldrich, MO, US) solution (0.005 % (v/v)) for 3 min for PLL coating. PLL is a polycation with positively charged amino groups that rapidly adsorbs onto alginate containing negatively charged carboxyl groups through electrostatic interactions [22]. This electrostatic interaction results in the formation of a PLL coating on the surface of the calcium alginate gel. PLL coating on calcium alginate gels has been shown to be safe *in vivo* [23].

### 2.5 Preparation of collagen gels for loading of hydrogel microspherical carriers

The collagen solution was prepared by mixing collagen acidic solution (IAC-50, KOKEN, Tokyo, Japan), Hanks' Balanced Salt Solution 10x (H1641-500ML, Sigma-Aldrich, MO, US), sodium hydrogen carbonate (198–01315, FUJIFILM Wako Pure Chemical Corporation, Osaka, Japan), HEPES (348–01372, DOJINDO LABORATORIES, Kumamoto, Japan), sodium hydroxide (190–14565, FUJIFILM Wako Pure Chemical Corporation, Osaka, Japan), and deionized water. Then, washed hydrogel microspherical carriers were loaded in the prepared collagen solution. Additionally, the collagen gel loaded with hydrogel microspherical carriers was prepared by gelatinizing the collagen solution by incubating it at 37 °C for 30 min. Previous studies have demonstrated the biocompatibility of collagen gel *in vivo* [20].

### 2.6 Observation of hydrogel microspherical carriers

To fluorescently visualize the alginate, fluorescein-5-thiosemicarbazide (FTSC, 76863–28-0, Life Technologies Corporation, CA, US)-modified sodium alginate was added to the non-labeled sodium alginate at 20 % (w/w). The concentration of sodium alginate at the observation was eventually adjusted to 2.5 % (w/v). To fluorescently visualize BVEs, BVEs were labeled by DilC_18_(5) (DiD,127274–91-3, Setareh Biotech, LLC, OR, US). Collagen gels and PLL were also fluorescently modified with Alexa Fluor^TM^ 568 NHS ester (A20003, Thermo Fisher Scientific, MA, US). Alexa Fluor™ 568 NHS ester was mixed with collagen solution and PLL at a volume ratio of 1:200. The NHS ester covalently binds to amino groups within the collagen gels and PLL, resulting in fluorescent labeling. The fluorescence-modified hydrogel microspherical carriers were observed by the confocal microscope. Note that only one of the collagen gels and PLL was stained at the time of observation, respectively.

### 2.7 Measurement of release ratio of FSNs

During the release experiments of FSNs from hydrogel disk-shaped carriers and hydrogel microspherical carriers, 1 mL of 5 mM calcium chloride solution was spread to the hydrogel carriers formed on the dish. To evaluate the released FSNs, the fluorescence intensity of FSNs from the supernatant of the hydrogel carriers after ultrasound irradiation was measured by a fluorescence spectrophotometer (FP-8550, JASCO Corporation, Tokyo, Japan). The supernatant of hydrogel carriers without FSNs was used as a blank. The release ratio of FSNs was calculated by dividing the obtained fluorescence intensity by the fluorescence intensity of the initial FSNs amount in the hydrogel disk-shaped carriers and hydrogel microspherical carriers, respectively. Note that the fluorescence intensity of the initial FSNs in the hydrogel disc-shaped carrier and hydrogel microsphere-shaped carrier was measured by degrading calcium alginate gel with alginate lyase[20].

### 2.8 Statistical analysis

Sample analyses were performed using analysis of Student's *t*-test. A value of ∗ p < 0.05 or ∗∗p < 0.01 was considered significant. In the graphs, error bars indicate standard deviation.

## 3 Results

### 3.1 Fabrication of ultrasound irradiation device

**Fig. 2:**
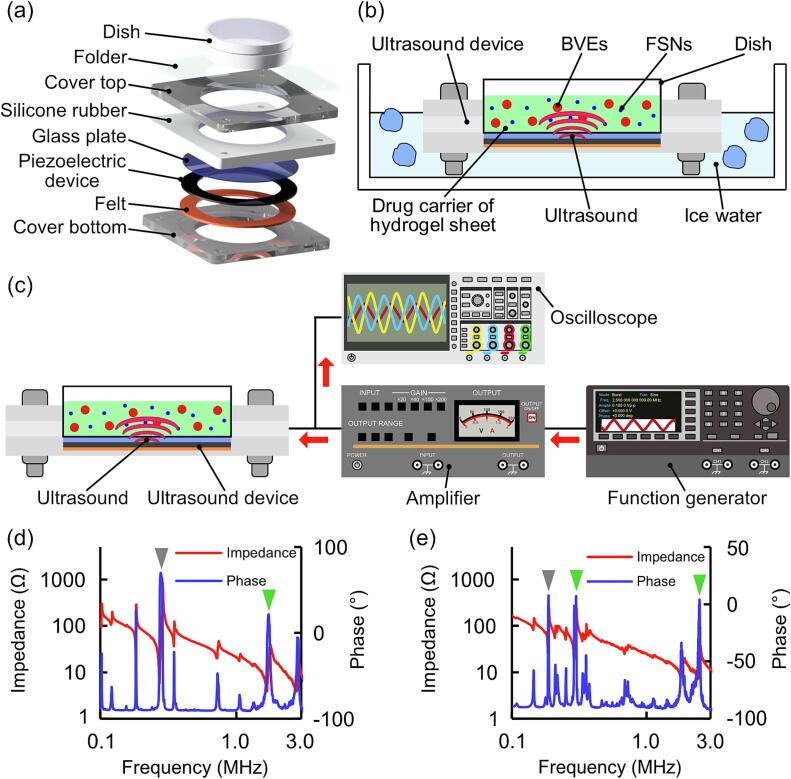
Experimental setup and evaluation of ultrasound irradiation device. (a) Components of ultrasound irradiation device. (b,c) Schematic illustration of drug release by hydrogel carrier using the ultrasound irradiation device. (b) Cooling of the ultrasound irradiation device and (c) peripheral equipment during ultrasound irradiation. (d,e) Resonance frequency measurements of ultrasound irradiation devices with (d) 0.5 and (e) 2.0 mm thick glass plate. Green and gray arrows indicate guaranteed acoustic pressures of 400 kPa and acoustic pressures less than 400 kPa, respectively.

An ultrasound irradiation system was constructed to investigate whether the inclusion of BVEs in a hydrogel carrier was effective for drug release using ultrasound stimulation. The ultrasound irradiation device (Fig. 2a) consists of a transducer with a piezoelectric element bonded to the glass and sandwiched between felt and silicone rubber to allow vibration without fixation. During ultrasound irradiation, to prevent the device's heat generation from affecting BVEs, experiments were conducted with the device immersed in cold water with ice (Fig. 2b). The ultrasound irradiation system consisted of a function generator, amplifier, oscilloscope, and ultrasound irradiation device. The ultrasound frequency and applied voltage were controlled by the function generator. The voltage was amplified by the amplifier before being supplied to the ultrasound irradiation device. An oscilloscope observed the voltage and current values flowing through the ultrasound irradiation device (Fig. 2c). The resonance frequency of the ultrasound irradiation device was varied by changing the thickness of the constituent glass, and the resonance frequency was measured using an impedance analyzer (Fig. 2d, e). The fabricated ultrasound irradiation device exhibited multiple resonance points between 0.1 and 3 MHz. The resonance frequency of the ultrasound irradiation device used in the experiment was selected to be guaranteed at 400 kPa stably, considering the strength of the acoustic pressure to be irradiated.

### 3.2 Acoustic pressure irradiated on hydrogel carriers

**Fig. 3:**
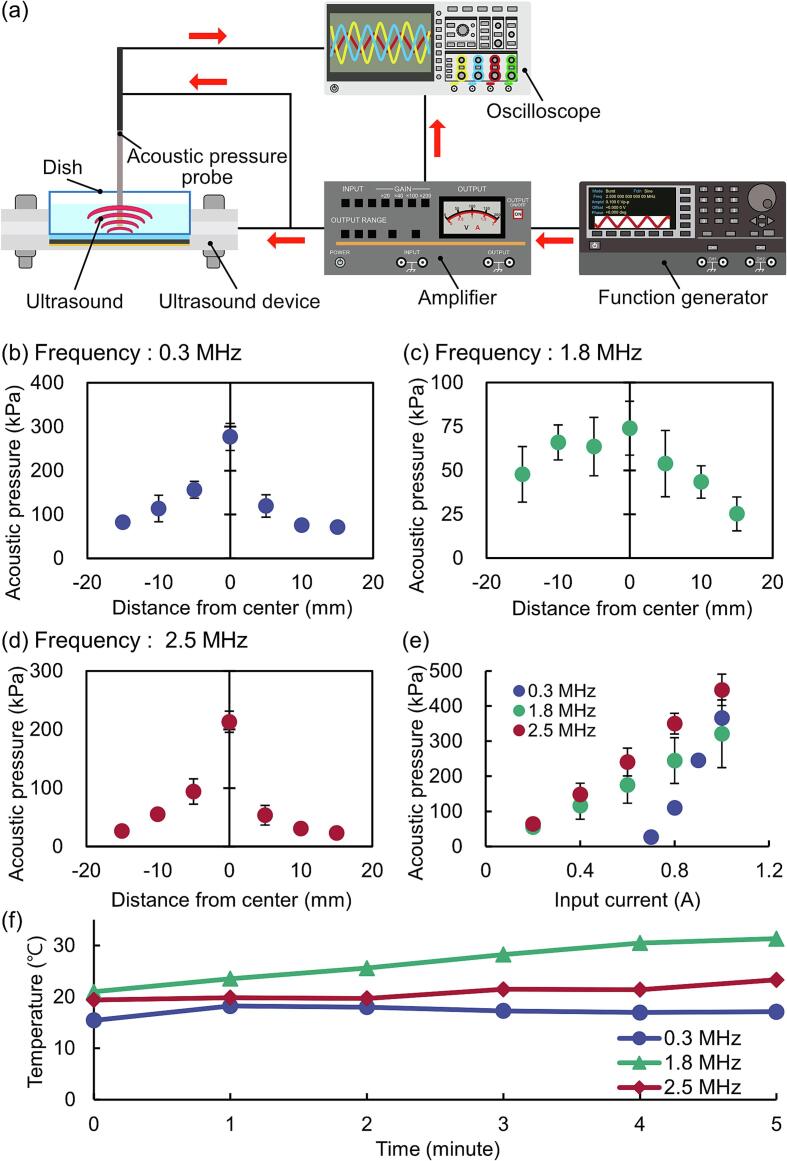
Acoustic pressure measurement of the ultrasound irradiation device and observation of temperature changes. (a) Experimental setup of the acoustic pressure measurement. (b-d) Acoustic pressure distribution was measured in the dish at frequencies of (b) 0.3, (c) 1.8 and (d) 2.5 MHz. (e) Relationship between applied current and acoustic pressure at the center of the dish. (f) Temperature changes in the dish during ultrasound irradiation. Error bars: mean ± S.D., *n* = 5.

When irradiating ultrasound within the body, it is necessary to control the output of acoustic pressure to a level that maintains the safety of biological tissues [24]. Previous findings on ultrasound irradiation to living bodies have confirmed that ultrasound irradiation to general living bodies, such as blood vessels, is safe as far as the spatial-peak temporal-average intensity (*I_SPTA_*) is less than 720 mW/cm^2^ [25]. Also, to mitigate the thermal effects of continuous ultrasound irradiation, we decided to irradiate ultrasound waves with a duty ratio of 0.5. Based on these conditions, an acoustic pressure of approximately 415 kPa is safe at a duty ratio of 0.5 from supplementary materials (Fig.S1). Therefore, the output was adjusted to achieve a maximum acoustic pressure of 400 kPa. To obtain the target average acoustic pressure, the acoustic pressure distribution of the fabricated ultrasound irradiation device was evaluated (Fig. 3a).

After defining the irradiation acoustic pressure and waveform conditions, the irradiation frequency was investigated. Frequency closely relates to cavitation generation. Cavitation generated by ultrasound irradiation is feared to cause damage to the biological tissues [26]. Consequently, medical ultrasound usually utilizes frequencies around 1.5–3.0 MHz, where cavitation is suppressed [27], [28], [29]. Meanwhile, the use of ultrasound with a frequency higher than several MHz is not practical because of the large attenuation in deep biological tissue [30]. Therefore, we selected the resonance frequencies around 1.5–3.0 MHz (∼1.8 MHz, 2.5 MHz) from the resonance frequencies of the device as the ultrasound irradiation frequencies. Note that, 1.8 and 2.5 MHz ultrasound have been demonstrated *in vivo* [31], [32], making it possible to expect safety and bioactivity. Additionally, the ultrasound frequency of 0.3 MHz, with one order of magnitude lower than the frequency, was also employed as a control.

Then, the acoustic pressure distribution with applied current to the fabricated ultrasound irradiation device was measured (Fig. 3b–d). The acoustic pressure of the center of the dish was maximum. This is because the center of the dish tends to oscillate due to the fixing of the edge of the dish. Furthermore, the relationship between the applied current and acoustic pressure (Fig. 3e) revealed a proportional increase in acoustic pressure with increasing current. Thus, the current was adjusted so that the acoustic pressure was 400 kPa at the center of the dish. Temperature measurements during ultrasound irradiation under these conditions confirmed the temperature is kept below 37 °C, a temperature that does not affect cells thermally. (Fig. 3f).

### 3.3 Evaluation of the effectiveness of BVEs

**Fig. 4:**
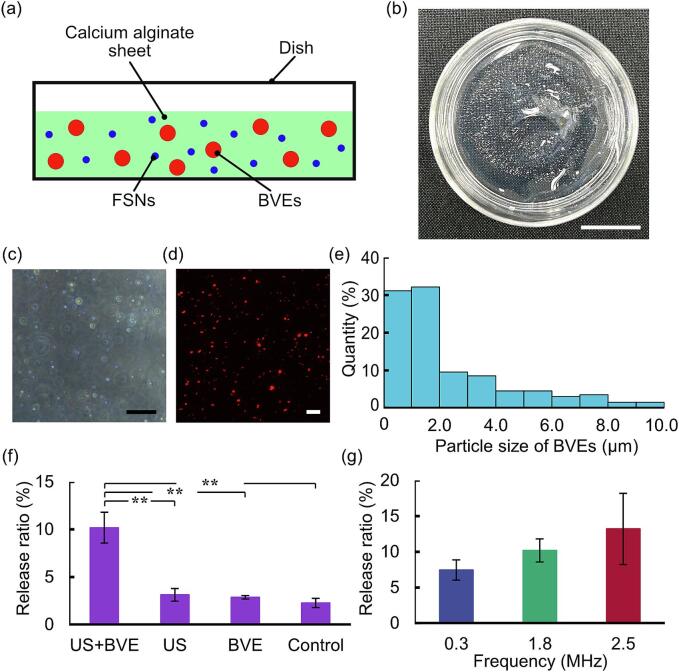
Release of fluorescent silica nanoparticles (FSNs) by ultrasound irradiation of hydrogel disk-shaped carriers with BVEs. (a) Components of hydrogel disk-shaped carrier in the dish. (b) Macroscopic image of the hydrogel disk-shaped carrier. (c,d) BVEs encapsulated in a hydrogel disk-shaped carrier were observed by (c) phase-contrast and (d) confocal microscopes. (e) The size distribution of BVEs in the hydrogel disk-shaped carrier. (f) The effects of ultrasound irradiation (US) and BVEs on the release ratio of FSNs were evaluated. (g) The effects of various frequencies of ultrasound irradiation on the release ratio of FSNs were evaluated. Scale bars of (b) = 10 mm, (c, d) = 15 μm. Error bars: mean ± S.D., *n* = 3. **p < 0.01.

In this study, microbubbles with shells were employed as BVEs, which can stably hold gas to enhance the acoustic responsiveness of the hydrogel carrier. BVEs were stable in hydrogels due to the shell of the lipid membrane. To confirm the response of the BVEs-encapsulated hydrogel carriers to ultrasound stimulation, hydrogel disk-shaped carriers were fabricated (Fig. 4a, b) for release experiments of FSNs. In order to verify the encapsulation of BVEs within the hydrogel, the BVEs within the hydrogel were observed by phase-contrast microscopy (Fig. 4c). Since these BVEs were trapped at varying depths, a histogram of the particle size distribution was obtained by a Z-stack image by confocal microscopy (Fig. 4d, e). The histogram showed that the average particle size was 2.4 µm ± 2.2 µm.

In order to evaluate the release ratio of the FSNs, the amount of the released FSNs was measured with a fluorescence spectrophotometer. For a demonstration of the synergistic effect of BVEs and ultrasound irradiation, the ratio of the released FSNs from the hydrogel disk-shaped carriers was evaluated using four conditions: ultrasound (US) and BVEs, US, BVEs, and a control (Fig. 4f). Note that the control was no BVEs encapsulated and no ultrasound irradiation. The ultrasound at 1.8 MHz for 5 min. with the maximum acoustic pressure at the dish center fixed at 400 kPa with a duty ratio of 0.5 were irradiated to the hydrogel disk-shaped carriers. From these results, the US + BVEs (= 10.2 %) and the US (=3.1 %) conditions increased the release amount of FSNs compared to the control condition (= 2.3 %). Furthermore, the proposed condition with BVEs and ultrasound irradiation (US + BVEs) exhibited a significantly higher release ratio of FSNs compared to the other conditions (^**^p < 0.01). The proposed condition was approximately 4.5 times higher than the condition with control. These results indicated that the combined effect of ultrasound and BVEs enhanced the release performance of the hydrogel carriers.

### 3.4 Evaluation of acoustic responsiveness by ultrasound frequency

To evaluate the frequency responsiveness of BVEs, the influence of varying the irradiated ultrasound frequency on release efficiency was measured. As release experiments of FSNs, hydrogel disk-shaped carriers containing BVEs were irradiated by ultrasound at three frequencies of 0.3, 1.8, and 2.5 MHz. The ratio of released FSNs (RR_0.3_, RR_1.8,_ and RR_2.5_) was defined from 0.3, 1.8, and 2.5 MHz frequencies of the irradiated ultrasound, respectively (Fig. 4g). These ultrasounds were adjusted to an acoustic pressure of 400 kPa with a duty ratio of 0.5 and were irradiated for 5 min. The average of RR_2.5_ is the highest, 1.8 times higher than RR_0.3_. Comparing RR_1.8_ and RR_2.5_, the average of RR_2.5_ is higher than that of RR_1.8_. These results indicated that the release ratio of FSNs from hydrogel carriers containing BVEs depends on the frequency of ultrasound irradiation.

### 3.5 Application of hydrogel microspherical carriers

**Fig. 5:**
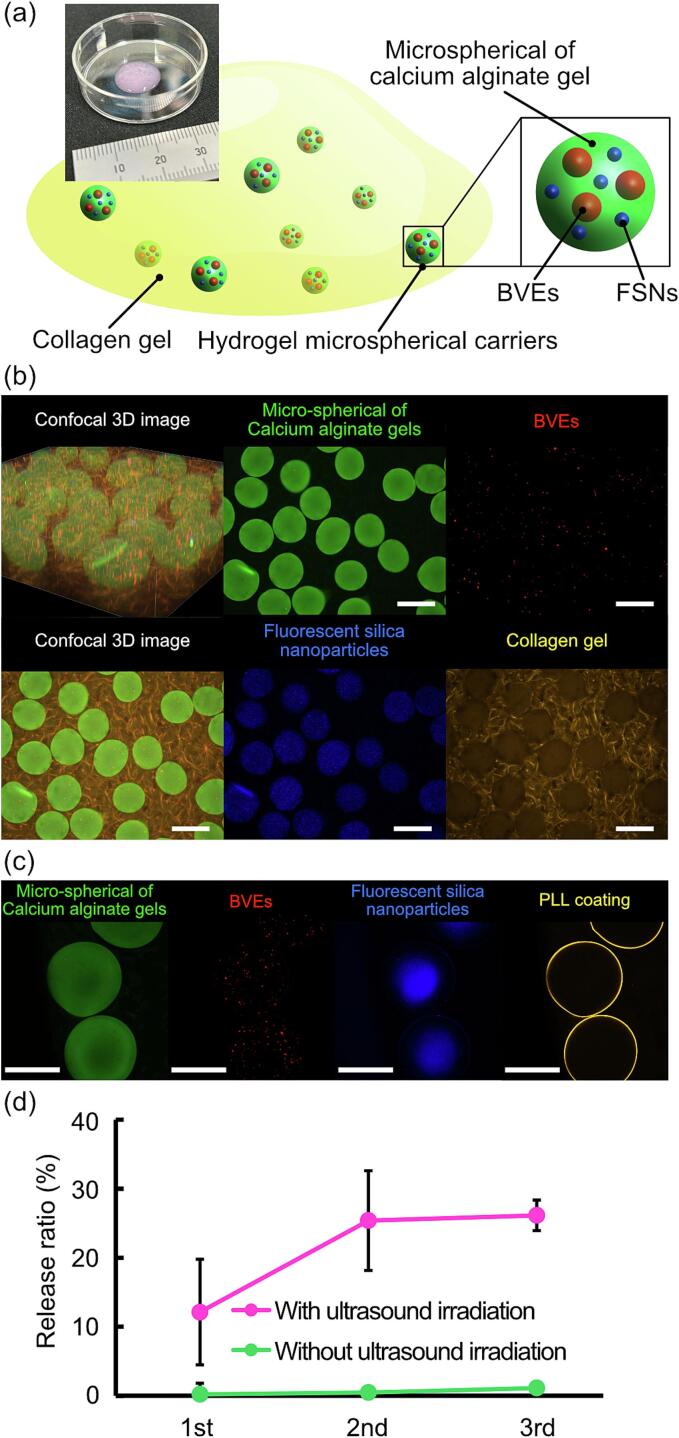
Demonstration of drug release with the hydrogel microspherical carrier for clinical applications. (a) Concept of the hydrogel microspherical carrier. (b) Confocal images of hydrogel microspherical carriers loaded on collagen gel. Microspherical calcium alginate gels, BVEs, FSNs, and collagen gel were stained with green, red, blue, and yellow, respectively. (c) Confocal image of hydrogel microspherical carriers coated by PLL (yellow). (d) Repeated release evaluation of FSNs from the PLL-coated hydrogel microspherical carriers by multiple ultrasound irradiation. Scale bars of (b, c) = 300 μm. Error bars: mean ± SD, *n* = 3.

For *in vivo* implantable DDS, a drug-releasing material implanted flexibly in the body has been devised using hydrogel microspherical carriers [20], [33]. Regarding the acoustic responsiveness demonstrated in this study, the release of the FSNs by ultrasound irradiation in the shape of hydrogel microspherical carriers was evaluated for its potential as a clinical application. In order to prevent movement during ultrasound irradiation and observation, the hydrogel microspherical carriers were loaded in a collagen gel (Fig. 5a). To verify the appropriate encapsulation of the BVEs and the FSNs into hydrogel microspherical carriers, the calcium alginate gel, BVEs, FSNs, and collagen gel were fluorescently stained and observed in green, red, blue, and yellow under a confocal microscope, respectively (Fig. 5b). For *in vivo* practicality, hydrogel microspherical carriers were coated with PLL, which was expected to provide long-term stable encapsulation [34], successfully suppressing the release of the FSNs in the steady state without ultrasound stimulation. Focusing on the PLL coating (yellow), the microparticle carriers were observed by confocal microscopy (Fig. 5c). Moreover, the difference in release of the FSNs with and without ultrasound irradiation was evaluated for the drug release performance of the PLL-coated hydrogel microspherical carriers. Similar to previous experiments, the average acoustic pressure of the ultrasound irradiation was 400 kPa with a duty ratio of 0.5. Additionally, the repeated responsiveness of the hydrogel microspherical carriers was confirmed by performing three cycles of ultrasound irradiation for 5-min. (Fig. 5d). Focusing on the first release, the release ratio of the US (12 %) improved significantly compared to that of without US (0.2 %). The differences in release ratio between US and control were also observed in repeated ultrasound irradiation. This indicates that the PLL-coated hydrogel microspherical carriers release the FSNs by ultrasound stimulation on demand. Moreover, focusing the control without ultrasound stimulation, the final release ratio was suppressed to 1.1 %, demonstrating the ability of PLL coating to control the release of the FSNs under steady-state conditions. Therefore, on-demand drug release in response to ultrasound is possible even at the micro size.

## 4 Discussion

In this study, BVEs were encapsulated in hydrogel as a drug release enhancer to fabricate hydrogel carriers with acoustic responsiveness and biocompatibility. The combined effect of ultrasound and BVEs improved the drug release performance of the hydrogel carriers (Fig. 4f). The irradiation of different ultrasound frequencies (Fig. 4g) suggested that the BVEs had frequency-dependent characteristics. In previous studies of DDS with acoustic responsiveness [18], drug models were released under different ultrasound outputs and irradiation durations with the same frequency. This means a need for knowledge on evaluating drug release performance by irradiating ultrasound of various frequencies. This is because the resonance frequency of ultrasound irradiation depends on the structure and materials of the transducer. Therefore, drug release by ultrasound irradiation at different frequencies requires transducers with the desired resonance frequency. Meanwhile, we fabricated transducers as a single rigid body by pressurizing and adhering glass to a piezoelectric element (Fig. 2a). By varying the thickness of the constituent glass, the transducers with different intrinsic vibration frequencies were designed (Fig. 2d, e), enabling irradiation of various frequencies. Consequently, the response of the hydrogel carrier was evaluated with various ultrasound frequencies from kHz to MHz bands.

Experiments were conducted at three frequencies in this study. For medical applications, the acoustic intensity during ultrasound irradiation is essential for biocompatibility. For drug release from hydrogel carriers [35] using low-frequency ultrasound (kHz range), the large size of the yielded cavitation from high amplitude [36] can cause rapid thermal action or shock waves [37], [38], [39], risking damage to biological tissues. Based on the threshold of cavitation generation obtained from acoustic pressure and frequency [40], three frequencies were chosen under the acoustic pressure of 400 kPa. 1) 0.3 MHz ultrasound at 400 kPa is in the region of high cavitation occurs. 2) 1.8 MHz and 3) 2.5 MHz ultrasounds at 400 kPa are in the boundary region where cavitation occurs. The experimental results indicated that RR_2.5_, which is in the frequency range with suppressed cavitation, shows a higher release ratio than RR_0.3_, which is in the frequency range with rich cavitation (Fig. 4g). These results suggest a relationship between the characteristics of BVEs and the release ratio.

Hence, we focus on the resonance characteristics of the BVEs. Since this experimental system is a composite material encapsulating microbubbles coated with a lipid membrane in a hydrogel, comprehensive calculations are difficult. Therefore, the simplest system, the Minnaert’s equation, was used. The resonance frequency, *f*, of BVEs having frequency dependence [41] can be derived from Minnaert’s equation [42],

(1) $$f=\frac{1}{2\pi R}\sqrt{\frac{3\gamma P}{\rho }}$$

where *R*, *γ*, *P*, and *ρ*, are a bubble radius, a specific heat ratio, a pressure around the bubble, and a density of the external fluid, respectively. Note that the specific heat ratio is taken as the polytropic coefficient of perflubutane, which is the main component of the BVEs [43], [44], [45]. From equation (1), the resonance frequency of the BVEs is obtained to be ∼ 2.4 MHz. Comparing the release ratio at each frequency, RR_2.5_ showed the highest release ratio. Regarding the average release ratio, RR_2.5_, RR_1.8_, and RR_0.3_ were the highest in that order (Fig. 4g). Therefore, the release ratio improved as the irradiated ultrasound frequency approached the theoretically calculated BVEs resonance frequency. This suggests that 2.5 MHz ultrasound resulted in the release of more nanoparticles trapped in the three-dimensional hydrogel network structure compared to other frequencies. That is, the hydrogel carrier using BVEs proposed in this study has the potential to release drugs more efficiently by using the MHz band closer to the resonance frequency of BVEs than the kHz band below the resonance frequency of BVEs. Since the ultrasound of our output (∼400 kPa, duty ratio of 0.5) allowed a safe *in vivo* test to be irradiated, we show the realization of hydrogel carriers combining acoustic responsiveness and biocompatibility. Furthermore, to enhance the biocompatibility of our hydrogel carrier by reducing the risk of immune reactions, several options are available: medical-grade low-endotoxin sodium alginate can be used for the calcium alginate gel [46], and atelocollagen, which exhibits low antigenicity *in vivo*, can be used for the collagen gel [47]. Regarding the physical properties, since calcium alginate hydrogel containing BVEs behaves predominantly elastically rather than viscously [48], the elastic behavior of the hydrogel would contribute to the vibration characteristics of the BVEs. Therefore, the properties of the BVEs can be characterized by the types and physical properties of hydrogels.

In the hydrogel carriers proposed in this study, the ratio of drug released by a single ultrasound irradiation was ∼ 4.5 times that of spontaneous slow release (Fig. 4f). This gradual sustained release is controllable by increasing the number or duration of ultrasound irradiation [18]. Focusing on the materials, ultrasound in the hydrogel disk-shaped carriers should be reflected by the BVEs located near the bottom of the dish, resulting in a potentially reduced release ratio (Fig. 4). The release ratio could be optimized by changing the shape of the hydrogel carriers. Hydrogel microspherical carriers are one of the optimal shapes because of their large specific surface area. The results (Fig. 5) showed that the release ratio of hydrogel microspherical carriers between US + BVEs and control was about 13 times higher than that of the hydrogel disk-shaped carriers between US + BVEs and control. *In vivo* applications with micro-sized hydrogel carriers [20] are also expected due to allowing for a high degree of freedom in the area to be administered. By reducing the scale, the surface of the hydrogel microspherical carriers could be electrically coated with PLL stably, consequently preventing drug leakage. In the future, the combination of our hydrogel microspherical carriers and smaller hydrogel carriers [49], [50], [51] is expected to expand the use of drug carriers *in vivo*, such as blood vessels. Thus, when hydrogel carriers are administered *in vivo*, the temperature is maintained at the body temperature of 37℃, which is slightly higher than the temperature conditions during ultrasound irradiation in this experiment (Fig. 2f). Here, considering ultrasound attenuation in biological tissues due to temperature, attenuation has been confirmed to decrease as temperature increases within the range of 4℃ to 37℃ [52], suggesting that our hydrogel carrier encapsulating BVEs should be no issues regarding ultrasound penetration *in vivo*. Meanwhile, since ultrasound attenuation in biological tissues increases with higher frequencies, the appropriate selection of ultrasound frequency according to the target tissue is also suggested to be important.

The gradual release of the drug by several ultrasound irradiations can be used effectively. For treatments requiring continuous administration, including adeno-associated virus-mediated gene delivery [5], [53] and insulin administration for diabetic patients [54], gradually releasing drug carriers is desirable. Unlike micelles [55] and liposomes [56], which release all the drugs by disrupting the lipid membrane, the hydrogel carriers proposed in this study have the advantage of releasing the drug gradually on demand. Therefore, the number of injections can be reduced by ultrasound irradiation, thus reducing the burden on the patient. Theranostics, where diagnosis and treatment are performed simultaneously [57], [58], require technology that releases drugs on demand in response to diagnosis. Our device can be a powerful tool for such medical technology. Combining these technologies with the hydrogel carriers proposed in this method is expected to develop *in vivo* devices that enable the potential of on-demand drug release.

## 5 Conclusions

This study reported a system that releases FSNs from BVEs-encapsulating hydrogel carriers through ultrasound irradiation controlled at acoustic pressures meeting *in vivo* safety standards. The resonance characteristics of BVEs were confirmed by irradiating at various ultrasound frequencies of 0.3 MHz, 1.8 MHz, and 2.5 MHz. In addition, we fabricated hydrogel microspherical carriers for clinical applications, demonstrating the release of FSNs control through PLL coating and repeated responsiveness to ultrasound stimulation. This system demonstrates hydrogel carriers capable of on-demand drug release in response to ultrasound under conditions guaranteeing *in vivo* safety.

## CRediT authorship contribution statement

**Ryuto Yamakawa:** Conceptualization, Investigation, Methodology, Writing – original draft, Visualization. **Hiroaki Onoe:** Methodology, Visualization, Writing – review & editing. **Yuta Kurashina:** Conceptualization, Methodology, Funding acquisition, Supervision, Writing – review & editing.

## Declaration of competing interest

The authors declare that they have no known competing financial interests or personal relationships that could have appeared to influence the work reported in this paper.
