# Supplementary material for: Hydrogel carrier with bubble vibration enhancer for ultrasound-triggered drug release
Source: Ultrason Sonochem. 2024 Nov 22;112:107173. doi: 10.1016/j.ultsonch.2024.107173 (PMC11635015; doi:10.1016/j.ultsonch.2024.107173)
Supplement: Supplementary Data 1 [file mmc1.docx]

Supplementary Materials

**Hydrogel carrier with bubble-vibrated enhancer for ultrasound triggered**

Ryuto Yamakawa^1^, Hiroaki Onoe ^2^, and Yuta Kurashina^1*^

^1^ Division of Advanced Mechanical Systems Engineering, Tokyo University of Agriculture and Technology, 2-24-16 Nakacho, Koganei-shi, Tokyo 184–8588, Japan

^2^ Faculty of Science and Technology, Keio University, 3-14-1 Hiyoshi, Kohoku-ku, Yokohama, 223-8522, Japan

*Corresponding author: Yuta Kurashina

E-mail: kurashina@go.tuat.ac.jp

**Table of Contents:**

S1. *I_SPTA_* calculation method

**S1. *I_SPTA_* calculation method**

Assume that the acoustic pressure waveform obtained from the hydrophone is as shown in Fig. S1. This waveform is represented over a time range *T* as follows:


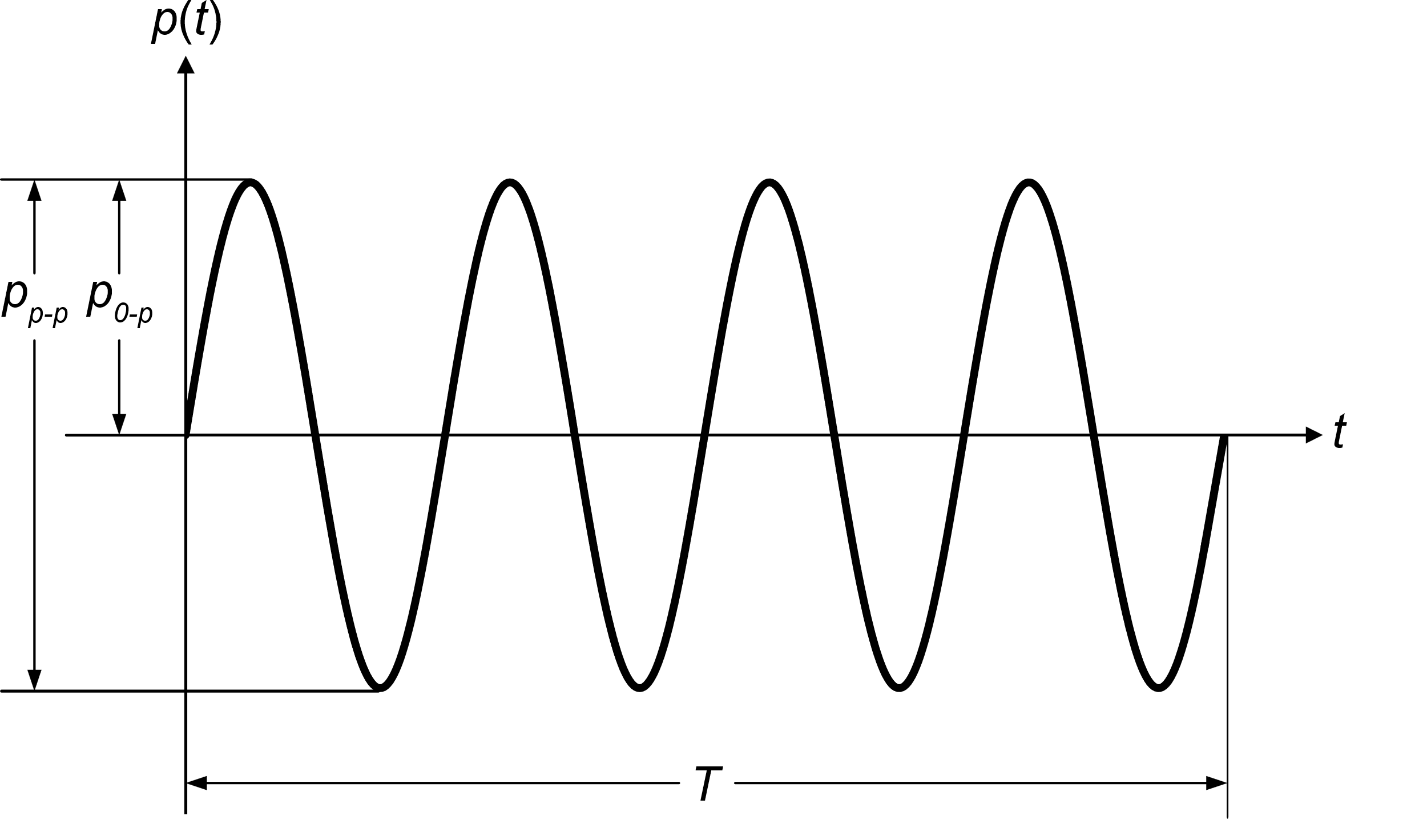


Fig. S1. Acoustic pressure waveform

$P\left( t \right)=p_{0-p}\sin2\pi ft$ (1)

Then, the squared function is:

$P^{2}\left( t \right)={p_{0-p}}^{2}\sin^{2}2\pi ft$ (2)

Rewriting in terms of peak-to-peak value, since *p*_0_*_-p_* = *p_p-p_*/2, equation (2) becomes:

$P^{2}\left( t \right)=\frac{{p_{p-p}}^{2}}{4}\sin^{2}2\pi ft=\frac{{p_{p-p}}^{2}}{8}\left( 1-\cos4\pi ft \right)$ (3)

Now, focusing on the time average of *P*^2^(*t*) denoted as ⟨*P*^2^(*t*)⟩ the second term in the parentheses of equation (3) is a periodic function, so its time average becomes zero. Therefore, the acoustic intensity I is:

$I=\frac{1}{\rho c}\left\langle P^{2}\left( t \right) \right\rangle=\frac{{p_{p-p}}^{2}}{8\rho c}$ (4)

*I_SPTA_* can be calculated from equation (4) using the *p_p-p_* at the position where the acoustic pressure is maximum. Furthermore, equation (4) assumes continuous wave ultrasound over the time range *T*. Therefore, for burst waves, it is necessary to consider the duty cycle, resulting in:

$I_{SPTA}=\frac{{p_{p-p}}^{2}}{8\rho c}Duty$ (5)
